# An axolotl limb regeneration-inspired strategy to enhance alveolar bone regeneration

**DOI:** 10.1016/j.bioactmat.2025.02.020

**Published:** 2025-02-19

**Authors:** Rongpu Liu, Guifang Wang, Li Ma, Guangzheng Yang, Sihan Lin, Ningjia Sun, Jiajia Wang, Huijing Ma, Xinquan Jiang, Wenjie Zhang

**Affiliations:** a Department of Prosthodontics, Shanghai Ninth People's Hospital, Shanghai Jiao Tong University School of Medicine, Shanghai, China; b College of Stomatology, Shanghai Jiao Tong University, Shanghai, China; c National Center for Stomatology, National Clinical Research Center for Oral Diseases, Shanghai, China; d Shanghai Key Laboratory of Stomatology, Shanghai Engineering Research Center of Advanced Dental Technology and Materials, Shanghai, China; e Department of Oral Implantology, Pudong New District Oculopathy Odontopathy Dispensary, Shanghai, China; f Faculty of Dentistry, Oral & Craniofacial Sciences, King's College London, London, UK

**Keywords:** Alveolar bone regeneration, BMP-2, Channel structure, Prrx1^+^ stem cell, Axoltol

## Abstract

Guided bone regeneration (GBR) is widely applied in implant dentistry, employing barrier membranes to create an osteogenic space by preventing gingival tissue ingrowth. However, this method does not enhance the osteogenic capacity of osteoblasts, limiting sufficient bone volume in larger defects. Inspired by axolotl limb regeneration, abundant soft tissue-derived stem cells mobilized to the defect may facilitate comprehensive osteogenesis within a BMP-2-enriched environment. We developed a biomimetic channel system (BCS) to promote alveolar bone regeneration, using channel structures to activate gingival-derived stem cells under a BMP-2-enriched biological barrier. In a cell-tracing mouse model, Prrx1^+^ stem cells demonstrated a critical role in BMP-2-induced subcutaneous osteogenesis. Sequencing and histological analyses revealed that channel structures significantly enhance soft tissue cell proliferation and migration. Attributable to the biological barrier, BCS applications markedly improved bone formation in beagle mandibular defects. These results suggest a novel osteoinductive strategy for alveolar bone regeneration that functions without a traditional barrier membrane.

## Highlights

• Cell-tracing mouse model uncovered the pivotal role of Prrx1^+^ stem cells in BMP-2-induced osteogenesis.
• Channel structures promoted the proliferation and migration of cells within infiltrated soft tissues.
• The biomimetic channel system (BCS) significantly improved alveolar bone regeneration outcomes.
• BMP-2-enriched biological barrier exhibited superior osteogenic efficacy compared to traditional barrier membranes.

## Graphical abstract

**Figure:**
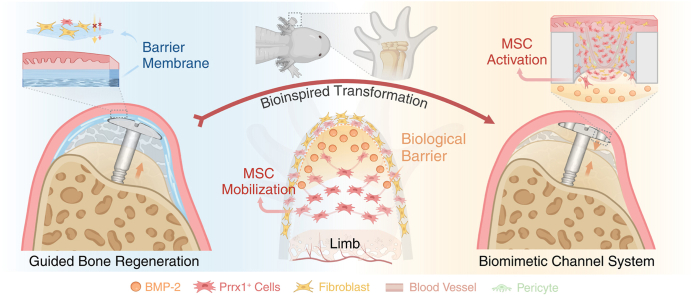

## 1 Introduction

Periodontitis, trauma, and prolonged tooth loss can lead to alveolar bone defects, negatively affecting facial aesthetics and masticatory function [[1], [2], [3]]. Clinically, GBR is commonly employed for augmenting local bone deficiencies in the alveolar ridge [4,5]. The principle of GBR involves using a barrier membrane to prevent the rapid ingrowth of fibroblasts from the gingival side, enabling bone tissue to gradually fill the defect from beneath [6,7]. Supra-alveolar bone regeneration in the severe defect possesses a significant challenge [2], where tenting screws can be employed to maintain the osteogenic space [[8], [9], [10]]. However, recruitment of skeletal stem cells to the defect site is typically slow and limited, presenting challenges for achieving adequate bone volume. In clinical practice, osteogenic factors such as bone morphogenetic protein-2 (BMP-2) are widely used to increase the osteoinductivity of bone substitutes and facilitate the repair of alveolar bone defects [11]. BMP-2 recruits mesenchymal stem cells in vivo and drives their differentiation into osteoblasts, promoting new bone formation [[12], [13], [14]]. In response to the growing demand for bone regeneration, advanced techniques such as micro- and nano-carrier-based sustained release systems, immune microenvironment modulation, and co-delivery of growth factors have been developed to optimize both the release kinetics of BMP-2 and its osteogenic environment [[15], [16], [17], [18]]. However, direct synergistic strategies for BMP-2 to specifically boost the osteogenic efficacy of stem cells remain limited.

**Fig. 1:**
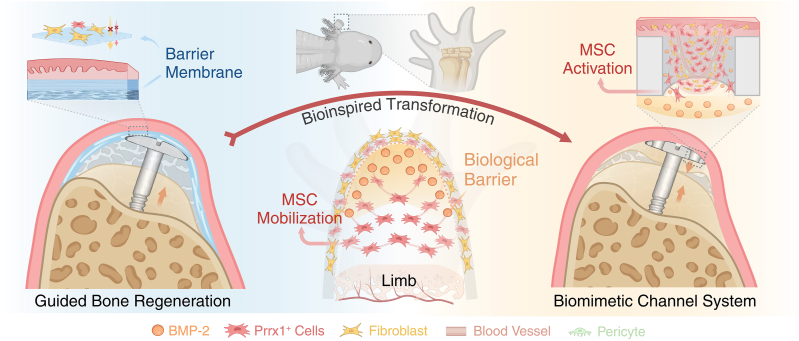
**SchematicshowingtheBMP-2-based biomimetic channel system for alveolar bone regeneration**. In traditional GBR procedures, a barrier membrane isolates gingival tissue, creating an osteogenic space that allows a gradual bottom-up alveolar bone regeneration process. Inspired by axolotl limb regeneration, complete bone regeneration occurs as mobilized stem cells contribute to osteogenesis within a BMP-2-enriched environment. Therefore, we developed the BCS that promotes osteogenesis by channel-activating gingival-derived stem cells under a BMP-2-enriched biological barrier. This innovative osteoinductive strategy enables simultaneous bidirectional osteogenesis from both the top and bottom of defect area, thereby enhancing alveolar bone regeneration. Illustration created with BioRender.

Research on the regenerative capabilities of amphibians has provided valuable insights into human regenerative medicine [19,20]. Remarkably, axolotls can completely regenerate limb bones after traumatic amputation [21]. Recent studies using live imaging and single-cell sequencing have revealed that injury response factors are highly expressed at the amputation site, activating Prrx1^+^ stem cells [[22], [23], [24]], which has been identified as critical contributors to craniofacial and limb development in mammals [25,26]. During axolotl limb regeneration, these Prrx1^+^ stem cells demonstrate high proliferative and migratory activity, serving as a key contributor to blastema formation [27,28]. Notably, BMP-2 is continuously expressed on the soft tissue side of the blastema, creating a BMP-2-enriched environment that maintains Prrx1^+^ stem cells in the regeneration of complete bone segments while also preventing excessive fibroblast infiltration [23,29,30]. Inspired by the limb regeneration process in axolotls, we developed a biomimetic channel system (BCS) that leverages the osteoinductive properties of BMP-2. The central concept is to promote alveolar bone regeneration by using a BMP-2-enriched biological barrier in conjunction with abundant activated stem cells. First, we demonstrated that BMP-2 primarily recruits Prrx1^+^ cells for osteogenesis in a cell-tracing mouse model, with osteogenic efficacy depending on the initial quantity of Prrx1^+^ cells. Building on prior research showing the influence of channel structures on cell behavior [[31], [32], [33], [34]], we conducted RNA sequencing and histological staining to investigate their activation effects on soft tissue cells, particularly in promoting cell proliferation and migration. In subcutaneous and gingival transplantation models, we further demonstrated the synergistic effects of BMP-2 and channel structure on the ectopic recruitment of Prrx1^+^ stem cells for osteogenesis. After validating these concepts, we proposed loading BMP-2 into bone substitutes to create a biological barrier, eliminating the need for traditional barrier membranes. This approach aimed to recruit and induce Prrx1^+^ stem cells from gingival tissue for osteogenesis, thereby maintaining the designated osteogenic space. Additionally, we incorporated channel structures into tenting screws and implants to activate gingival tissue cells, thereby expanding the pool of activated stem cells and amplifying the osteogenic effect of BMP-2. Compared to traditional GBR, the BCS effectively promoted alveolar bone regeneration on the gingival side, leading to significant vertical bone volume restoration in beagle mandibular defects. Our BCS enables simultaneous “bidirectional osteogenesis” from both the soft tissue and bone tissue sides of the defect area, highlighting its promising potential for clinical application (Fig. 1).

## 2 Materials and methods

### 2.1 Experimental design

All experiments were independently replicated at least three times. The sample size (n) for each experiment is indicated in the figure legends. For in vivo studies, animals were randomly assigned to experimental groups. All surgical procedures and sample analyses were conducted in a blinded and consistent manner. Statistical methods are detailed in the 'Statistical Analysis' section.

### 2.2 Materials

Calcium phosphate cement (CPC) granules and recombinant human BMP-2 (rhBMP-2) were supplied by REBONE Biomaterials (China). Resorbable bone substitutes (Bio-Oss®) and resorbable collagen membranes (Bio-Gide®) were purchased from Geistlich (Switzerland). Silk fibroin was extracted from *Bombyx mori cocoons* according to established protocols [31] and prepared into a 50 mg/mL silk solution. Porous silk sponges were fabricated via freeze-drying, crosslinked in methanol for 2 h, and then sterilized by autoclaving at 121 °C for 20 min. The silk sponges were then trimmed to a diameter of 5 mm and a height of approximately 3 mm, and subsequently coated with 0.3 mg/mL rhBMP-2 for in vivo applications.

### 2.3 Animals

Wild-type C57BL/6 mice, SD rats and beagles were provided by the Animal Laboratory Center of Shanghai Ninth People's Hospital Affiliated with Shanghai Jiao Tong University. *Prrx1-cre; R26R*^*tdTomato*^ mice were obtained from Cyagen Biosciences (China). For this study, 8-week-old mice and rats were used for subcutaneous and subgingival transplantation, and 18-month-old beagles were used for alveolar bone augmentation. All experimental protocols were approved by the Institute for Laboratory Animal Research of the Ninth People's Hospital Affiliated with Shanghai JiaoTong University, School of Medicine (approval number: SH9H-2020-A227-1).

### 2.4 Pseudotime analysis

**Fig. 2:**
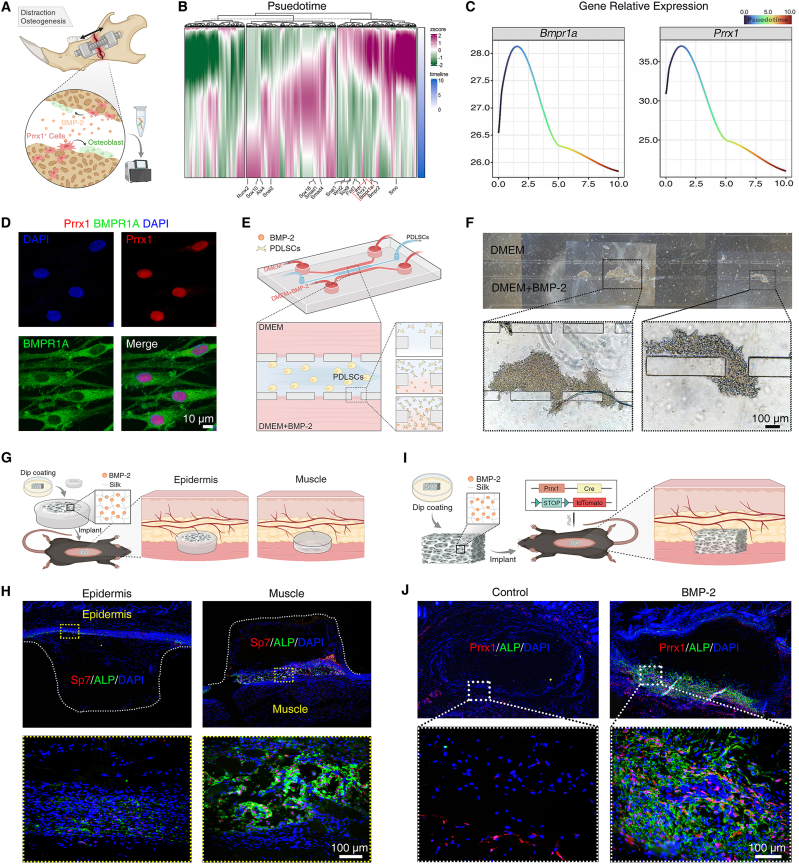
**Prrx1**^**+**^**stem cells demonstrated a critical role in BMP-2-induced osteogenesis**. (**A**) Schematic illustration of a mouse distraction osteogenesis model. (**B**) Genes of osteogenic stem cells sorted by time of peak expression during jaw regeneration.(**C**) Expression patterns of *Bmpr1a* and *Prrx1*. (**D**) Co-localization of Prrx1 and BMPR1A in hPDLSCs. (**E**) Schematic diagram of cell migration experiment using a three-channel microfluidic chip. (**F**) After hPDLSCs adhered in the central channel, culture medium containing 200 ng/mL BMP-2 was perfused in the upper channel while the standard culture medium in lower channel; cell migration is observed after 8 h. (**G**) Schematic of semi-open silk sponges subcutaneously implanted with open sides facing either the epithelial or muscular region in Mice. (**H**) Sp7/ALP co-staining was performed to assess osteogenic effects in the localized region on day 7. (**I**) Schematic of silk sponges for dorsal subcutaneous transplantation in *Prrx1-cre; R26R*^*tdTomato*^ mice. (**J**) ALP immunofluorescence staining was utilized to trace the osteogenic differentiation of BMP-2-recruited Prrx1^+^ stem cells. n = 3 biological replicates. Illustration created with BioRender.

Pseudotime analysis was performed as previously described [35,36]. The RNA-seq data were sourced from the GEO database [37], and the pseudotime analysis was based on the bulk-RNA of bone, cartilage and skeletal progenitors in mandibular distraction osteogenesis (POD5 to POD15). All expressed genes (TPM >1 in any sample) were used to separate samples by PCA. Then, the euclidean distances of adjacent samples along the development trajectory were calculated to produce developmental time unit values, scaled from 0 to 10. For each gene, the Z-scaled expression values were interpolated into 500 points along its pseudotime metric using the loess function in R (v4.4.1) to allow for smooth continuous comparisons. PCA on Z-scaled expression values was performed for each gene. The genes were ranked based on their peak expression timing using the Atan2 function in R (v4.4.1). This ranking was then utilized to generate the pseudotime heatmap and phasigrams presented in Fig. 2B and C.

### 2.5 Isolation and culture of hPDLSCs

Teeth were extracted from three patients (aged 18–25 years) for orthodontic purposes, stored in sterile PBS, and transferred to the laboratory within 10 min. All procedures were approved by the Ethical Committee of Shanghai Ninth People's Hospital. The modified outgrowth method was applied to culture human periodontal ligament stem cells (hPDLSCs). Briefly, periodontal ligament was gently isolated and cut into pieces of approximately 1 mm, and a sterile coverslip with vaseline (Sigma-Aldrich, USA) in its four corners was placed on top of the pieces. The vaseline at the corners increased the contact area between the tissues and the bottom of the culture dish, thereby promoting the outgrowth of hPDLSCs. Cells were cultured in Dulbecco's Modified Eagle Medium (DMEM; HyClone, USA) containing 10 % fetal bovine serum (FBS; Gibco, USA), 100 units per mL penicillin-streptomycin (HyClone, USA). Cells at passages P3-P5 were used for cytological experiments.

### 2.6 Study of cell migration in vitro

Microfluidic chips were obtained from MesoBioSys (China). The chip substrate was designed with a three-channel structure, featuring numerous small pores along the channel walls to facilitate fluid exchange between the channels. hPDLSCs were seeded in the central channel at a density of 1 × 10^5 cells/mL. Once the cells adhered, the upper channel was continuously perfused with standard culture medium, while the lower channel was perfused with 200 ng/mL rhBMP-2 culture medium. After 8 h, cell migration was observed using an inverted microscope (Olympus, Japan).

### 2.7 Fabrication of PDMS-based composites

The PDMS substrate was prepared by mixing the elastomer and cross-linker (Dow Corning, USA) in a weight ratio of 10:1. The mixture was stirred vigorously with a spatula and degassed under vacuum to eliminate air bubbles. Subsequently, it was poured into molds and cured at 60 °C overnight to produce no-channel PDMS films, single-channel PDMS films, channel-array (3 × 5 channels) PDMS films, and PDMS cubes, with all channels having a diameter of 800 μm. The specimens were ultrasonically cleaned and sterilized by autoclaving before use. To facilitate the rapid removal of the scaffold material and minimize its impact on tissue ingrowth, a sol state of gelatin (30 mg/mL; Aladdin, China) loaded with rhBMP-2 (0.3 mg/mL) was injected into the channel of the single-channel PDMS film and subjected to reversible low-temperature crosslinking. To simulate the effect of BMP-2-loaded bone substitutes, a silk sponge was prepared within the cavity of the PDMS cube, followed by the injection of rhBMP-2 (0.3 mg/mL). For the semi-open silk sponge, a shallow pit with a diameter of 5 mm and a depth of 1 mm was fabricated on the no-channel PDMS film, and the silk sponge was then prepared within the pit. Details on the grouping and applications of the PDMS composites are provided in Supplementary Table S1.

### 2.8 Fabrication of titanium implants and tenting screws

Titanium implants (Φ 4 mm × 9 mm) and tenting screws (head: 4 mm × 6 mm; shaft: Φ 1.6 mm × 10 mm) were produced using gas-atomized Ti–6Al–4V powder (EOS, Germany), with an average particle size of 15–45 μm, as the printing substrate. CAD modeling was performed, followed by the production of titanium implants with radial channels and titanium tenting screws featuring channel-integrated heads using a direct metal laser melting (DMLM) machine (Concept Laser M2, GE Additive, Germany), all with a channel diameter of 1000 μm. The specimens underwent ultrasonic cleaning in acetone, ethanol, and distilled water. To enhance osseointegration, the surface of implants was modified with micro-arc oxidation (MAO) as described previously [38]. Briefly, implants were subjected to MAO in an electrolyte containing 4.5 g/L glycerophosphate disodium salt pentahydrate (C_3_H_7_Na_2_O_6_P·5H_2_O; Kelong, China) and 4.0 g/L sodium metasilicate nonahydrate (Na_2_SiO_3_·9H_2_O; Sinopharm, China). All specimens were sterilized by autoclaving before use.

### 2.9 Fabrication of PEEK implants

PEEK implants (Φ 4 mm × 9 mm) were fabricated using fused deposition modeling (FDM) with PEEK filament (Polymics, USA) on a 3D printer (KB3/KB3M, 3d pro, China), featuring radial channels with a diameter of 1000 μm. After printing, the specimens were sequentially washed in an ultrasonic bath with ethanol and distilled water, then dried at 60 °C in a vacuum oven. To enhance bioactivity, implants were subjected to surface sulfonation as described previously [39]. Briefly, the specimens were sulfonated by immersion in 98 % concentrated sulfuric acid while stirring magnetically at room temperature for 10 min. Subsequently, the specimens were soaked in ddH_2_O for 10 min to remove any residual sulfuric acid, treated at 120 °C for 5 h to eliminate excess sulfur, washed twice with ddH_2_O, and allowed to dry at room temperature. All specimens were sterilized by autoclaving before use.

### 2.10 Subcutaneous transplantation model in mice and rats

After anesthesia with isoflurane, a longitudinal incision was made on the dorsal area to separate the subcutaneous tissue. The silk sponges or PDMS-based composites were then inserted subcutaneously through the incision, which was subsequently sutured. Both mice and rats underwent the same experimental procedure to establish the model.

### 2.11 Subgingival transplantation model in rats

Under isoflurane anesthesia, an 8-mm sagittal incision was made at the center of the hard palate in rats, and the periosteal flaps were elevated bilaterally. Dissection was performed along the surface of the maxilla toward its lateral aspect, expanding the space between the attached gingiva and the maxilla. A single-channel PDMS film, either loaded with or without rhBMP-2, was inserted into the created space and positioned with its base in contact with the alveolar bone, as described in a previous study [40]. Finally, the soft tissue was repositioned, and the palatal incision was carefully sutured.

### 2.12 RNA sequencing

No-channel and channel-array PDMS films were inserted subcutaneously in mice for 7 days. Total RNA was extracted from the subcutaneous tissue beneath no-channel PDMS films and from the channel structures of channel-array PDMS films using RNAiso Plus (TAKARA, Japan), designated as the Flat group and the Channel group respectively. The quantification, RNA-sequencing library construction and sequencing of all samples were conducted by CloudSeq Biotech (China). In brief, after purification, fragmentation and amplification, the high-quality reads were aligned to the reference genome with hisat2 software. Then, guided by the Ensembl gtf gene annotation file, cuffdiff software (part of cufflinks) was used to get the FPKM as the expression profiles of mRNA, and fold change and p-value were calculated based on FPKM, differentially expressed mRNA was identified. GO and Pathway enrichment analysis were also performed based on the differentially expressed mRNAs. The volcano plot, GO chord plot and GO bubble plot were established with Hiplot Online Tools (https://hiplot.com.cn/home/index.html).

### 2.13 Histology/immunofluorescence staining

hPDLSCs were fixed with 4 % polyformaldehyde at room temperature for 15 min and washed with PBS prior to immunofluorescent staining. For decalcified sections, samples were fixed in formalin and embedded in paraffin. Serial sections (5 μm) were prepared for H&E staining (G1120, Solarbio, China), Masson's trichrome staining (G1340, Solarbio, China), and immunofluorescence staining. In immunofluorescence staining procedure, the cells or decalcified sections were incubated overnight at 4 °C with primary antibodies: Prrx1 (ab211292, Abcam, USA), BMPR1A (ab264043, Abcam, USA), α-SMA (ab124964, Abcam, USA),CD31 (AF3628, R&D systems, USA), MyD88 (ab2064, Abcam, USA), ALP (AF2910, R&D systems, USA), Sp7 (ab22552, Abcam, USA) and OCN (23418-1-AP, Proteintech, China). Appropriate fluorescently labeled secondary antibodies (Abcam, USA) and DAPI (Sigma-Aldrich, USA) were applied afterward. The mounted sections were imaged using a confocal laser scanning microscope (CLSM, Leica TCS SP2, Germany). For non-decalcified sections, samples were fixed in formalin, dehydrated in gradient ethanol, and embedded in polymethylmethacrylate (PMMA; Sigma-Aldrich, USA). The samples were sectioned to a thickness of 150 μm using a saw microtome (SP1600, Leica, Germany) and polished to a final thickness of approximately 40 μm. These sections were stained with Goldner's trichrome staining (G3550, Solarbio, China) to observe osteogenesis.

### 2.14 EdU staining assay

For the EdU staining assay, mice were injected with EdU (50 mg/kg, ST067, Beyotime, China) intraperitoneally 8 h before subcutaneous tissue collection. Cell proliferation was detected using the Beyo-Click EdU Cell Proliferation Kit with Alexa Fluor 594 (C0078, Beyotime, China).

### 2.15 Alveolar bone augmentation with tenting screws in beagles

Under general anesthesia, a cylindrical defect (10 mm in diameter) was created in the posterior mandible using a dental handpiece and bur. To evaluate the effects of the tenting screw BCS on alveolar bone regeneration, traditional GBR surgery was employed as a control group. In the GBR group, a no-channel tenting screw was implanted at the center of the defect, with the height of its head aligned with the alveolar ridge. The defect was filled with a bone substitute composed of a 1:1 mixture of Bio-Oss® and CPC granules, which was then covered with a Bio-Gide® membrane. In the BCS group, a channel-integrated tenting screw and the bone substitute loaded with 0.30 mg/mL rhBMP-2 were used to support the osteogenic space, without the coverage of a barrier membrane.

### 2.16 Alveolar bone augmentation with implants in beagles

All premolars and first molars were extracted under general anesthesia, followed by a 3-month healing period. Initially, to preliminarily demonstrate the alveolar bone regeneration induced by the implant BCS, titanium implants were placed in the posterior mandible, with the channels filled with BMP-2-loaded silk sponge. The control group received silk sponge without the addition of rhBMP-2. Subsequently, to verify the role of gingival tissue in alveolar bone regeneration and to eliminate the radiative effects of titanium, PEEK implants were used to repair mandibular defects. Using a dental handpiece and bur, an 8 mm deep and 20 mm long block defect was created in the posterior mandible, and PEEK implants loaded with or without rhBMP-2 were subsequently placed in the defect.

### 2.17 X-ray and CT imaging for bone formation assessment

All beagles were sacrificed eight weeks post-surgery using overdose anesthesia. The mandible was dissected, and the sections containing the tenting screws and implants were carefully harvested. The samples were scanned with an X-ray imaging system (MultiFocus, Faxitron, USA) and Micro-CT (μCT50, Scanco Medical, Switzerland) to assess bone formation. In Micro-CT analysis, a cylindrical region centered on the defect site was selected as the region of interest (ROI) for 3D reconstruction and quantification of marginal bone level, new bone volume, bone volume/tissue volume (BV/TV), and biomaterial volume using Mimics (v26.0) and Avizo (v2020.1). Specifically, marginal bone level refers to the vertical distance measured from the lowest point of the alveolar crest surrounding the tenting screw shaft to the base of the mandibular defect.

### 2.18 Statistical analysis

Statistical analyses were performed using GraphPad Prism 10 statistical software (GraphPad, USA). All data were demonstrated as the means ± SD. Significant differences were calculated with unpaired *t*-test. *P* < 0.05 was considered as a statistically significant difference.

## 3 Results

### 3.1 BMP-2 recruited Prrx1^+^ stem cells for osteogenesis

BMPs bind to their receptors and activate specific signaling pathways, which induce stem cells to differentiate into osteoblasts. This process plays a key role in bone defect repair. Based on a recent RNA-sequencing study of mouse mandible regeneration [41], we explored the major type of stem cell induced by BMPs (Fig. 2A). This analysis revealed a sequential upregulation of genes associated with the BMP signaling pathway, including *Bmpr1a*, *Smad1*, *Smad4*, and *Runx2* (Fig. 2B and Fig. S1). Additionally, we observed that the mesenchymal cell marker gene *Prrx1* was synchronously expressed with *Bmpr1a* (Fig. 2C), suggesting that Prrx1^+^ stem cells are likely involved in mandible regeneration under the influence of endogenous BMP-2. To validate BMP-2's recruitment effect on Prrx1^+^ stem cells, we selected hPDLSCs as model cells. First, we confirmed the co-localization of Prrx1 and the BMP-2 receptor protein BMPR1A in hPDLSCs through immunofluorescence staining (Fig. 2D). Subsequently, we conducted the cell migration study using a three-channel microfluidic chip (Fig. 2E). Initially, hPDLSCs were seeded in the central channel and allowed to adhere, then perfused the culture medium. Under the recruitment of BMP-2, a significant number of hPDLSCs migrated to the interface between the central and lower channels after 8 h, indicating that Prrx1^+^ stem cells may serve as a cellular source for BMP-2-induced osteogenesis (Fig. 2F). To investigate the impact of tissue cell sources on BMP-2-based osteogenesis in vivo, semi-open BMP-2-loaded silk scaffolds were subcutaneously transplanted into C57BL/6 mice, with the open area facing either the epithelial or muscular tissue (Fig. 2G). Sp7^+^ and ALP^+^ early osteogenic cells with extracellular matrix in muscle group were significantly more than in epithelial group (Fig. 2H and Fig. S2). To further elucidate the different BMP-2-induced osteogenic effects at cellular level, we generated *Prrx1-cre; R26R*^*tdTomato*^ mice to trace Prrx1^+^ stem cells (Fig. 2I). The silk sponges with or without BMP-2 were implanted subcutaneously in mice, followed by ALP immunofluorescence staining on day 7. BMP-2 recruited a large number of Prrx1^+^ stem cells into the silk sponge, most of which co-localized with the osteogenic marker (ALP), and the osteogenic effect on the muscular side was notably superior to that on the epithelial side (Fig. 2J). We observed that the Prrx1^+^ stem cells in muscular side were initially more than in epithelial side, indicating that BMP-2 induced osteogenic effect is related to the initial quantity of Prrx1^+^ stem cells in the local soft tissue.

### 3.2 Channel structure upregulated gene expression in activate soft tissue cells

**Fig. 3:**
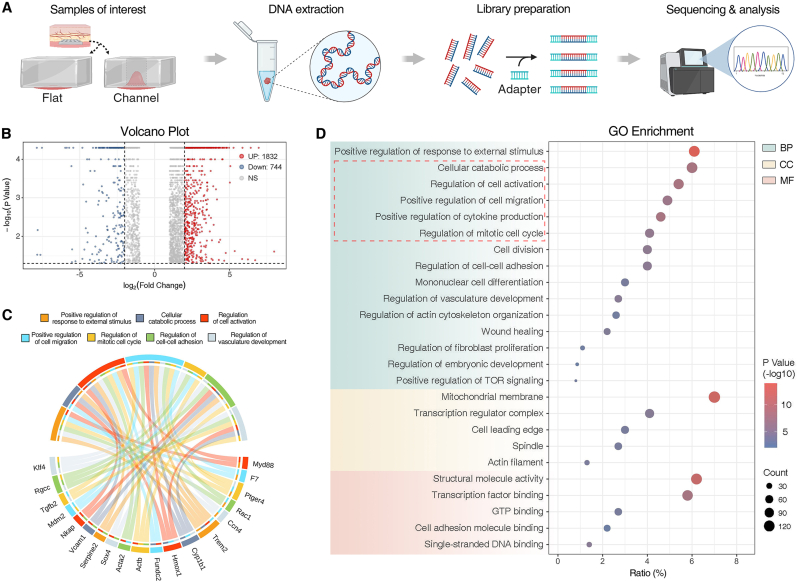
**RNA-seq of flat and channel group PDMS films after subcutaneous implantation into Mice**. (**A**) Overview of the procedure. Total RNA obtained from flat and channel group on day 7 were sequenced and analyzed. (**B**) Volcano plot showing DEGs by RNA sequencing. (**C** and **D**) GO chord (C) and bubble plots (D) display GO enrichment analysis of upregulated genes in channel group. n = 3 biological replicates. Illustration created with BioRender.

Regulating cell behavior through the physical cues provided by scaffold materials is a simple and effective tissue engineering strategy. To investigate the regulatory effects of channel structures on soft tissue cells, we implanted channel-array and no-channel PDMS films subcutaneously in mice and performed RNA sequencing on day-7 samples (Fig. 3A). Comparative clustering analysis of differentially expressed genes (DEGs) between the flat and channel groups revealed 1832 upregulated and 744 downregulated genes in the channel group, suggesting that channel structures induce significant transcriptomic changes in internal cells (Fig. 3B). Gene Ontology (GO) enrichment analysis showed that DEGs associated with response regulation to external stimuli and cell activation were enriched in the channel group. Furthermore, DEGs related to cellular metabolism, cell migration, cell cycle, and cell adhesion in biological processes (BP) suggested that channel structures may regulate diverse cellular behaviors. Using a GO chord diagram, we analyzed key upstream DEGs in these biological processes, identifying seven genes involved in multiple processes: Krüppel-like factor 4 (*Klf4*), regulator of cell cycle (*Rgcc*), beta-actin (*Actb*), heme oxygenase-1 (*Hmox1*), cytochrome P450 1B1 (*Cyp1b1*), and prostaglandin E2 receptor 4 (*Ptger4*). We also observed a significant upregulation of *MyD88*, potentially related to DEGs involved in response regulation to external stimuli and cell activation (Fig. 3C). The GO bubble plot indicated that the stimulatory effects of the channel structure on internal cells influenced proliferation, migration, and cellular metabolism, promoting vascularization within infiltrating tissue and generating embryogenesis-like biological processes. In terms of molecular function (MF), DEGs related to mitochondrial membranes and transcription regulator complexes indicated active cellular behavior [42]. DEGs in cell leading edges and actin filaments were linked to collective cell migration [43,44], while those related to spindles implied an increased frequency of mitotic events. Similarly, cellular component (CC) enrichment highlighted DEGs involved in cell adhesion and proliferative activity (Fig. 3D).

### 3.3 Activating effects of channel structure on soft tissue cells

**Fig. 4:**
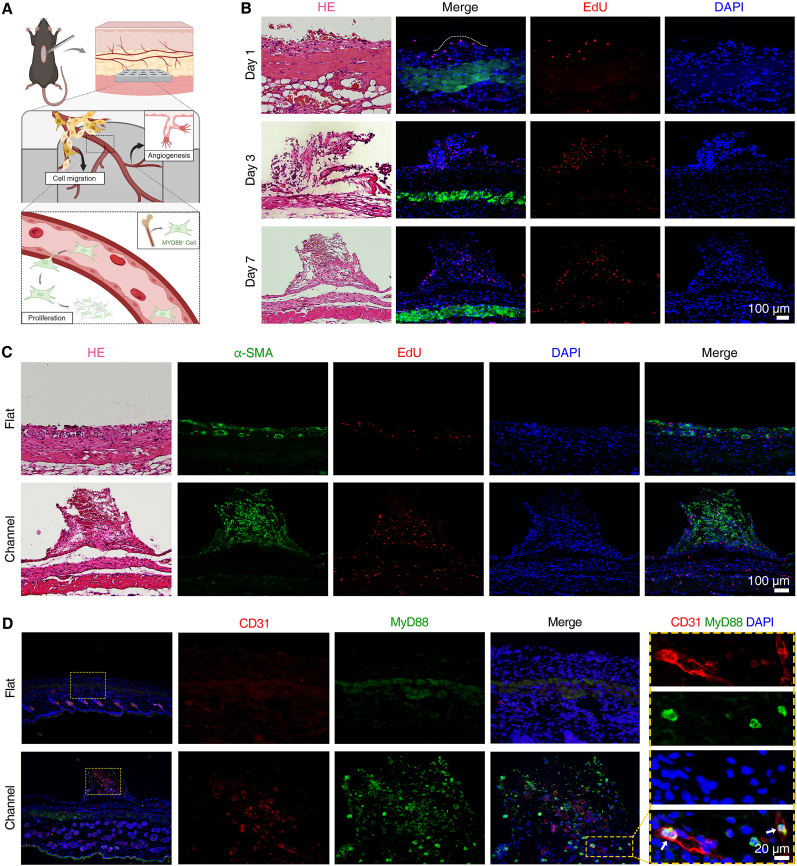
**Channel structure inducedingrowth of vascularized soft tissue with highly proliferative cells**. (**A**) Schematic illustration showing the channel structure facilitating rapid infiltration of vascularized soft tissue containing highly proliferative cells. (**B**) Hematoxylin-eosin (HE) and EdU staining in the channel group assessing the infiltration and proliferation of soft tissue cells in channel structures on days 1, 3, and 7. (**C**) α-SMA and EdU co-staining evaluating angiogenesis and cell proliferation in the surrounding soft tissue. (**D**) Spatial relationship between CD31 stained blood vessels and MyD88^+^ myeloid-derived cells on day 7. n = 4 biological replicates. Illustration created with BioRender.

To identify the activating effects of the channel structure on infiltrating cells, we subcutaneously transplanted the channel-array and no-channel PDMS films in mice and then validated the results using histological staining (Fig. 4A). After immune cells infiltration, newly formed soft tissue rapidly ingrew the channels on day 3, and filled the channel structure on day 7. For proliferative cells labeling, EdU solution was injected intraperitoneally before tissue collection. EdU staining showed the infiltrated tissue contains a significant number of proliferative cells during day 3–7 (Fig. 4B). Co-staining with α-SMA, we found significantly higher vascularization in channel group compared to flat group, suggesting the newly formed vessels supported the proliferative activity of perivascular cells (Fig. 4C). Based on the enrichment of biological processes related to mononuclear cell differentiation in the RNA sequencing results, we performed immunofluorescence staining for myeloid differentiation factor 88 (MyD88), and observed a significant presence of MyD88^+^ myeloid-derived cells distributed within the channel structure. Co-staining with CD31, MyD88^+^ cells were present within or surrounding the CD31 stained blood vessels (Fig. 4D). We speculate that the channel structure facilitates angiogenesis and recruits a population of myeloid-derived cells via the circulatory pathway, contributing to tissue regeneration within the channel structure [[45], [46], [47]].

### 3.4 Channel structure and BMP-2 synergically induced subcutaneous osteogenesis

**Fig. 5:**
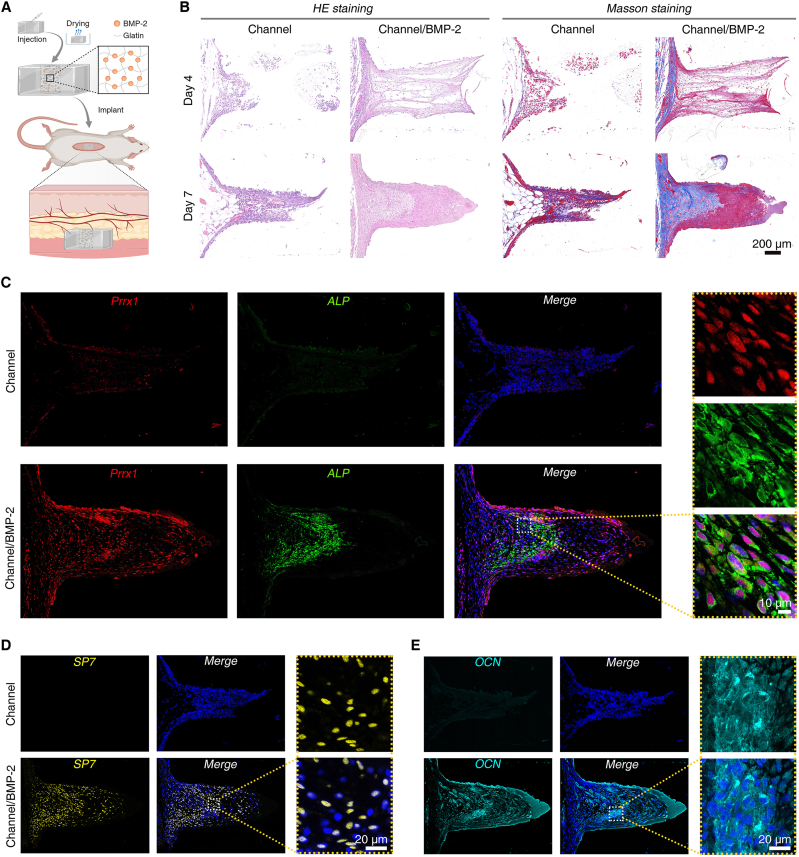
**Channel structure and BMP-2 inducedectopic recruitment of Prrx1**^**+**^**stem cells for osteogenesis**. (**A**) Schematic illustration of the single-channel PDMS film for dorsal subcutaneous transplantation in rats. (**B**) Evaluation of tissue infiltration and collagen formation within the channel structure using HE staining and Masson's trichrome staining on days 4 and 7. (**C**) Prrx1/ALP co-staining detects the osteogenesis of BMP-2 ectopically recruited Prrx1^+^ stem cells on day 7. (**D** and **E**) SP7 (D) and OCN (E) immunofluorescence staining for ectopic osteogenesis within channel structure on day 7. n = 4 biological replicates. Illustration created with BioRender.

Given that the recruitment and inductive effects of BMP-2 on Prrx1^+^ stem cells, along with the promoting effects of channel structures on cell proliferation and migration, the single-channel PDMS films loaded with BMP-2 were implanted subcutaneously in rats for evaluating their synergistic effects (Fig. 5A). HE staining was conducted to preliminarily explore the degree of infiltration and composition of newly formed tissue within the channels. Control group exhibited only a small number of deeply stained immune cells in channel structure on day 4, while the BMP-2 group showed a dense accumulation of immune cells and a mesh-like structure filling the channel lumen. Seven days post-operation, a weak bud-like tissue containing numerous immune cells was formed in channel group, whereas a robust and dense bud-like structure was developed in BMP-2 group. Corresponding Masson's trichrome staining results suggested that the amount of collagen fibers formed base within the BMP-2 group was significantly greater than that in the Control group, accounting for half-length of the bud-like tissue (Fig. 5B). To investigate the synergistic effects of BMP-2 and the channel structure on the induction of Prrx1^+^ stem cells for osteogenesis, immunofluorescence staining for Prrx1 and osteogenic markers was performed on day-7 samples. Under BMP-2 induction, a greater significant number of Prrx1^+^ stem cells were recruited into the channel structure, exhibiting a high density in areas of collagen fiber formation. Co-staining with ALP indicated that these soft tissue-derived Prrx1^+^ stem cells were undergoing osteogenic differentiation (ALP^+^) (Fig. 5C). Further SP7 and OCN immunofluorescence staining confirmed that BMP-2 induced a substantial number of cells to participate in osteogenesis within the channel, with these cells distributed throughout the entire lumen (Fig. 5D and E). These in vivo results demonstrated the synergistic effects of BMP-2 and the channel structure on the ectopic recruitment of Prrx1^+^ stem cells for osteogenesis.

### 3.5 Synergistic strategy applied in BCS model and subgingival osteogenesis

**Fig. 6:**
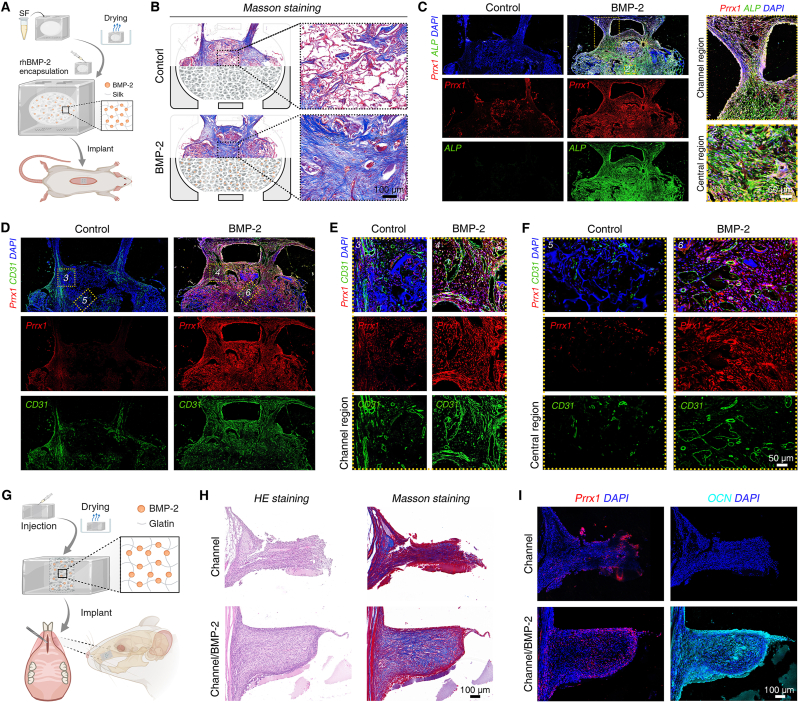
**Synergistic osteogenic effects of BMP-2 and channel structure in PDMS cube and subgingival tissue**. (**A**) Schematic illustration showing the PDMS cube implanted subcutaneously in rats as a BCS model. (**B**) Evaluation of collagen formation within the PDMS cube via Masson's trichrome staining on day 14. (**C**) Assessment of the sequential induction of stem cell infiltration and osteogenesis by BMP-2 and channel structures through Prrx1/ALP co-staining on day 14. (**D** to **F**) Whole-sample scan of CD31 staining on day 14 showing overall vascularization within the PDMS cubes (D), along with magnified views of the channel region (E) and the central region (F). (**G**) Schematic illustration of the single-channel PDMS film for subgingival transplantation in rats. (**H**) HE staining and Masson's trichrome staining were used to assess tissue infiltration and collagen formation within channel structures on days 4 and 7. (**I**) Prrx1 and OCN immunofluorescence staining for ectopic osteogenesis within channel structure on day 7. n = 4 biological replicates. Illustration created with BioRender.

Based on the synergistic effects of BMP-2 and channel structures in subcutaneous osteogenesis, we aim to continue exploring the feasibility of this synergistic strategy in upgraded BCS model and gingival tissue. PDMS cubes with surface channels and a large internal BMP-2-enriched region were fabricated as a research model for tenting screw BCS (Fig. 6A). Fourteen days post-operation, Masson's trichrome staining was conducted to study the infiltration of newly formed tissue within the PDMS cubes. BMP-2 induced a greater extent of vascularized soft tissue ingrowth along the channels compared to the control group (fig. S3, A and B), accompanied by significant material degradation and collagen fiber formation in the central region (Fig. 6B). To elucidate the osteogenic activity within biological barrier region, Prrx1 and ALP co-staining was performed. The results revealed that the BMP-2 group recruited a large number of Prrx1^+^ soft tissue stem cells, which underwent cell proliferation after the activation of channel structures (fig. S3 C), the osteogenic activity spread throughout the cavity of the PDMS cube (Fig. 6C). CD31^+^ blood vessels were both abundant in the channel regions of control and BMP-2 group, likely attributed to the angiogenic effects of the channel structures. Notably, BMP-2-enriched biological barrier influenced the collective cell migration behavior of the newly formed tissue, accompanied by extensive angiogenesis and enriched Prrx1^+^ stem cells and in central region (Fig. 6D–F). To extend the synergistic osteogenic strategy in gingival tissue, we subgingivally implanted single-channel PDMS films with or without BMP-2 in rats (Fig. 6G). HE and Masson's trichrome staining were performed to evaluate tissue infiltration and collagen formation within the channels on day 7. The results suggested that the depth of newly formed tissue infiltration was similar between the two groups, but the BMP-2 group exhibited a wider bud-like structure containing a significant amount of collagen fibers (Fig. 6H), containing a higher number of Prrx1^+^ stem cells than control group, accompanied by numerous OCN^+^ cells, suggesting that gingival tissue can participate in alveolar bone regeneration under the synergistic induction of the channel structure and BMP-2 (Fig. 6I).

### 3.6 Alveolar bone augmentation with tenting screw BCS in beagles

**Fig. 7:**
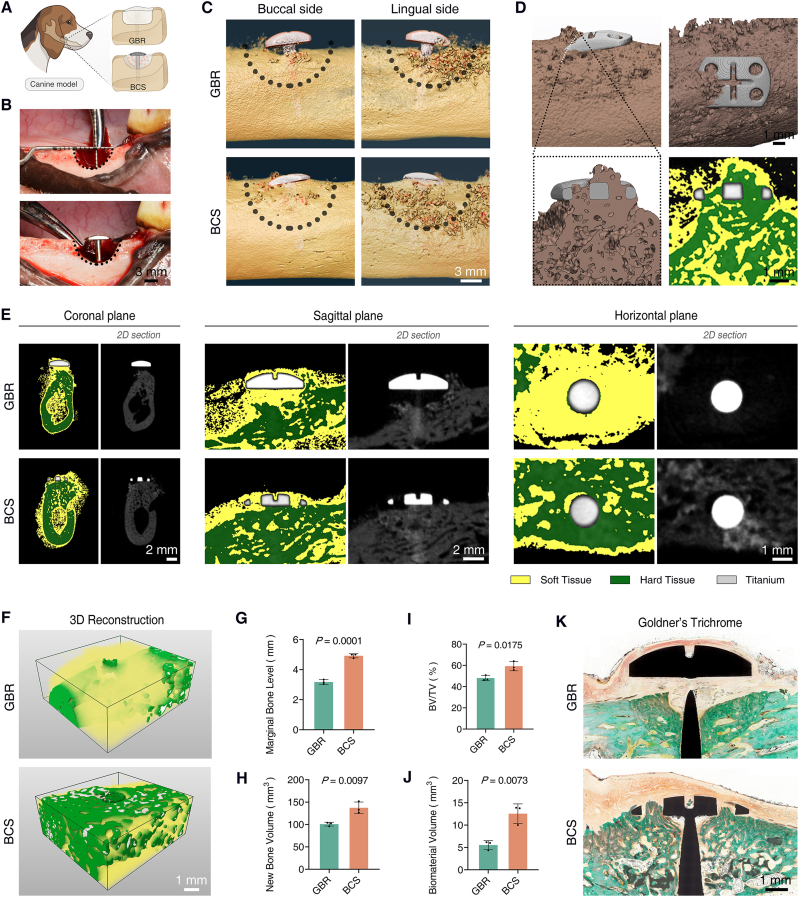
**Tenting screw BCS enhance**d **alveolar bone regeneration in canines**. (**A**) Schematic illustration showing the alveolar bone augmentation with GBR and BCS in beagles. (**B**) Intraoperative view of the mandibular defect. (**C**) Macroscopic view of CT reconstruction with the mandibular samples at eight weeks post-operation. (**D**) Macroscopic and sectional view of CT reconstruction with the tissue around channel structure in BCS group. (**E**) Three-dimensional cross-sectional view of mandibular samples. (**F**) Three-dimensional visualization of the soft tissue, bone tissue, and bone substitutes surrounding the tenting screws. (**G**) Statistics analysis of the marginal bone levels around tenting screws. (**H** and **I**) New bone volume and bone volume fraction (BV/TV) in the mandibular defects for evaluating newly formed bone tissue. (**J**) Retained biomaterial volume of bone substitutes for evaluating the stability of osteogenic space. (**K**) Goldner's trichrome staining for evaluating the alveolar bone augmentation around the tenting screws. n = 3 biological replicates, statistical differences were assessed using an unpaired *t*-test. Data are presented as means ± SD. *P* < 0.05 is considered as a statistically significant difference. Illustration created with BioRender.

To evaluate the application of tenting screw BCS in alveolar bone regeneration, 10-mm diameter semicircular defects were created in the mandibles of beagles. The defects were repaired with channel-integrated tenting screws and BMP-2-loaded bone substitutes, while the control group received conventional GBR with no-channel tenting screws and barrier membranes (Fig. 7A and B). Alveolar bone formation was assessed through Micro-CT imaging and reconstructions at eight weeks post-operation. Preliminary reconstruction results indicated that BCS significantly enhanced the osteogenesis of the alveolar bone defects, with the newly formed alveolar bone closely integrated with the screw heads and its height consistent with the surrounding alveolar ridge. In contrast, the GBR group exhibited a noticeable gap between the post-operative alveolar bone and the screw heads, along with significant displacement of the bone substitute (Fig. 7C and Fig. S4). CT reconstruction of the channel region in the BCS group revealed the significant osteogenesis surrounding the channel structure (Fig. 7D), indicating that the gingival tissue infiltrated into the channel structure participated the alveolar bone regeneration induced by BMP-2. To further compare the effects of the BMP-2-enriched biological barrier with conventional barrier membranes on establishing osteogenic extent, CT threshold segmentation was employed to differentiate between soft tissue, bone tissue, and tenting screws. Compared to GBR group, more alveolar bone tissue of BCS group formed in three-dimension sections. BCS nearly restored the alveolar ridge to its natural contour, while a significant soft tissue mass beneath the tent head was observed in the GBR group (Fig. 7E). Further three-dimensional reconstruction and bone quantification statistics around the tenting screws confirmed that BCS enhanced osteogenesis in defects, with higher reconstructed alveolar level and more stable osteogenic space (Fig. 7F–J). Goldner's trichrome staining also revealed that the BCS group achieved a greater volume of new bone, effectively promoting alveolar bone regeneration within the predetermined osteogenic space (Fig. 7K).

### 3.7 Alveolar bone augmentation with implant BCS in beagles

**Fig. 8:**
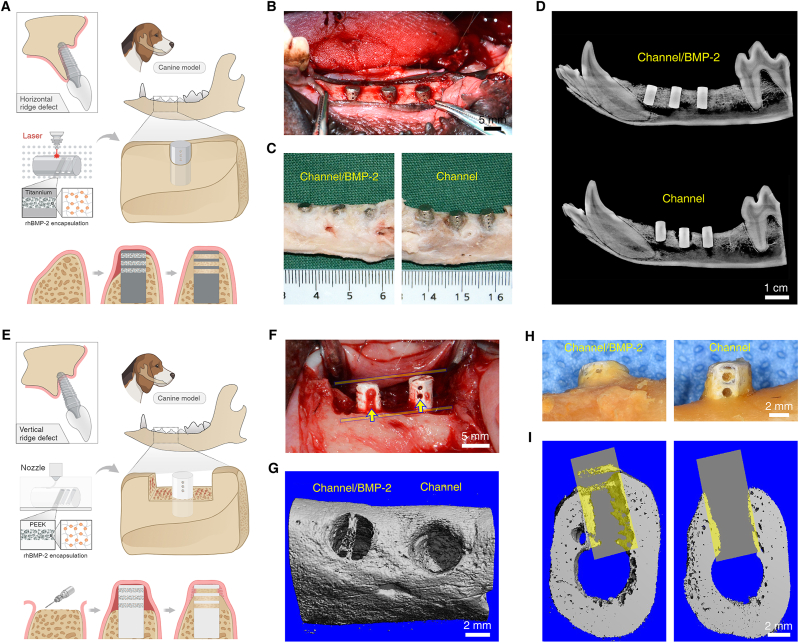
**Implant BCS enhance**d **alveolar bone regeneration in canines**. (**A**) Schematic illustration of the channel-integrated titanium implant implanted in beagles' mandible for bone augmentation. (**B**) Intraoperative view of the mandibular defect. (**C** and **D**) Macroscopic observations (C) and X-ray imaging (D) for assessing alveolar bone regeneration at eight weeks post-operation. (**E**) Schematic illustration of the channel-integrated PEEK implant implanted in mandibular defects for bone augmentation in beagles. (**F**) Macroscopic observation of the surgical site at eight weeks post-operation. (**G** to **I**) Macroscopic observations (G and H) and CT imaging (I) for evaluating the origin of newly formed bone at eight weeks post-operation. n = 3 biological replicates. Illustration created with BioRender.

Previous studies have demonstrated that channel-integrated implants can preserve the alveolar ridge height through inducing bone tissue ingrowth into channel structures [48]. To meet the growing demand for alveolar bone augmentation, we propose leveraging implant BCS to promote alveolar bone regeneration by harnessing the inductive effects of BMP-2 and channel structures on gingival tissue. Initially, implants loaded with or without BMP-2 were implanted into beagles' mandibular molar region, with one side of the channels adjacent to the bone wall and the other side exposed to gingival tissue (Fig. 8A and B). 8 weeks post-surgery, macroscopic observations indicated that BMP-2 significantly induced alveolar bone augmentation, with the openings of the channels completely filled with bone tissue, whereas the control group exhibited minor vertical resorption of alveolar bone (Fig. 8C). X-ray imaging corroborated these findings, showing a notably increased height of the alveolar ridge in BMP-2-loaded implants, with intimate integration between the implants and surrounding bone tissue (Fig. 8D). To further clarify the osteogenic potential of gingival tissue, we created a bulk bone defect in the canine mandible, measuring 8 mm in depth (Fig. 8E). The implants’ material was replaced with PEEK for avoiding radiopacity, and the openings at both ends of channels were kept exposed to the gingival tissue. Macroscopic observation showed that the channels in the BMP-2 group were filled with bone-like tissue, whereas the channels in the control group appeared empty (Fig. 8F). BMP-2 induced new bone tissue formation within the channel away from the defect surface, along with an elevation in alveolar ridge height (Fig. 8G–I). These in vivo results revealed the osteogenic contribution of gingival tissue to alveolar bone regeneration when using an implant BCS, which changed the conventional bottom-up osteogenic process.

## 4 Discussion

Extensive alveolar bone defects significantly impact the quality of patients' life and are typically addressed through GBR. However, GBR involves a slow, bottom-up osteogenic process, where the limited availability of mandibular stem cells hinders sufficient bone volume formation. Inspired by the limb regeneration process in axolotls, where abundant activated soft tissue stem cells can lead to remarkable bone regeneration in a BMP-2-enriched environment. Accordingly, we propose the BCS based on BMP-2's osteoinductive properties and the activation of channel structures, employing a biological barrier for alveolar bone augmentation instead of a conventional barrier membrane. Gingival tissue is induced to participate in bone regeneration at the superior margin of predefined osteogenic space, establishing a bidirectional osteogenesis process that enhances alveolar bone repair.

We presented the first evidence that BMP-2 recruits soft tissue-derived Prrx1^+^ stem cells for osteogenesis using the stem cell-tracing mouse model. Notably, the initial quantity of Prrx1^+^ stem cells significantly influenced the final BMP-2-induced osteogenic outcome. We speculate that enlarging the pool of Prrx1^+^ stem cells could lead to complete bone regeneration like the limb regeneration process in axolotls. In previous studies, Prrx1^+^ stem cells have been confirmed to participate in periodontal tissue regeneration [49,50]. Building on the activation of Prrx1^+^ stem cells under a BMP-2-enriched biological barrier, the biomimetic strategy effectively promoted alveolar bone regeneration in rat subgingival transplantation and beagle alveolar bone augmentation models.

Channel structures were incorporated into our BCS for enlarging the pool of activated stem cells. We employed bioinert and non-degradable biomaterials as matrix materials for constructing channel structures, thereby ensuring the long-term stability of the channel structures in vivo. Pre-validation via RNA-seq confirmed the activation of soft tissue cells by the channel structures, with various biological processes including cell proliferation, migration, and metabolism being stimulated. Additionally, we observed an upregulation of *Klf4* in the Channel group, which is a key "Yamanaka factors" regulating cell pluripotency [51]. Same upregulation is also evident in the activation of stem cells during limb regeneration in axolotls, promoting the proliferation and collective cell migration of stem cells through modulation of the mitotic cycle and intercellular adhesion processes [24]. Further histological analyses validated the positive effects of the channel structures on internal cell proliferation and migration. We also discovered that the channel structures enhance angiogenesis and recruit MyD88^+^ bone marrow-derived cells via circulatory pathways, likely facilitating the regeneration process through direct participation or mediation of cell activation [[45], [46], [47]].

Our bioinspired BCS innovatively enhanced alveolar bone regeneration under a BMP-2-enriched biological barrier. To demonstrate the feasibility of BCS model, we fabricated PDMS cubes with surface channels and a large internal region enriched with BMP-2. BMP-2 enrichment in the cube cavity facilitated the rapid ingrowth of Prrx1^+^ stem cells, with a substantial number occupying the PDMS cube for osteogenesis, rather than excessive, adverse fibroblasts. Combined with the activation effects of channel structures, the BCS model effectively induced intensive osteogenesis within the cube. In the same subcutaneous transplantation model, a higher number of Prrx1^+^/ALP^+^ double-positive cells were observed in PDMS cube samples on postoperative day 14 compared to Single-Channel PDMS Film samples on postoperative day 7. This indicates that BCS effectively and progressively recruited and induced a significant population of Prrx1^+^ stem cells to participate in osteogenesis.

Subsequently, we applied the tenting screw and implant BCS in canine alveolar bone augmentation models. Compared to traditional GBR, tenting screw BCS remarkably promoted alveolar bone formation without barrier membranes, obtaining higher reconstructed alveolar level and more stable osteogenic space. For implant BCS application, the synergistic induction of channel structures and BMP-2 resulted in increased alveolar ridge height, marking an upgraded application of channel-integrated implants from previous studies on alveolar ridge preservation. Overall, all channel structures fabricated from different materials exhibit significant osteogenic effects in synergy with BMP-2. Notably, recent studies have emphasized the critical regulatory role of extracellular matrix (ECM) stiffness in determining stem cell fate, which has been shown to promote osteogenic differentiation [52]. Therefore, future studies could explore the role of matrix mechanical properties in activating cells within channel structures and their synergistic interactions with BMP-2.

This study presents a novel osteoinductive strategy for alveolar bone regeneration that eliminates the need for barrier membranes, and amplifies BMP-2's regenerative effects through the activation of channel structures. The applications of BCS significantly accelerate alveolar bone regeneration via the bidirectional osteogenesis model, highlighting the clinical translational potential of this biomimetic strategy. Furthermore, factors such as the effects of additional cytokines, changes in mechanical interface, and loss of cell-cell contact may also activate stem cells [53,54]. It is expected that corresponding strategies combined with BMP-2's osteoinductivity could lead to the additional treatments for bone regeneration.

## 5 Conclusions

Inspired by axolotl limb regeneration, a BMP-2-enriched biological barrier was engineered to preserve the osteogenic space, with channel structures serving as efficient conduits for activated stem cells to enhance alveolar bone regeneration. By promoting cell proliferation and migration, channel structures facilitated BMP-2-mediated recruitment of numerous soft tissue-derived Prrx1^+^ stem cells for ectopic osteogenesis. In subsequent applications, the tenting screw BCS and implant BCS significantly enhanced bone formation on the gingival side of the predefined osteogenic region, highlighting an innovative biomimetic approach for alveolar bone augmentation that eliminates reliance on traditional barrier membranes.

## CRediT authorship contribution statement

**Rongpu Liu:** Writing – original draft, Methodology, Conceptualization. **Guifang Wang:** Writing – original draft, Methodology, Conceptualization. **Li Ma:** Writing – original draft, Visualization, Conceptualization. **Guangzheng Yang:** Visualization, Funding acquisition. **Sihan Lin:** Methodology, Data curation. **Ningjia Sun:** Methodology, Data curation. **Jiajia Wang:** Software, Investigation. **Huijing Ma:** Visualization, Data curation. **Xinquan Jiang:** Writing – review & editing, Supervision, Funding acquisition. **Wenjie Zhang:** Writing – review & editing, Funding acquisition, Conceptualization.

## Ethics approval and consent to participate

Wild-type C57BL/6 mice, SD rats and beagles were provided by the Animal Laboratory Center of Shanghai Ninth People's Hospital Affiliated with Shanghai Jiao Tong University. *Prrx1-cre; R26R^tdTomato^* mice were obtained from Cyagen (China). For this study, 8-week-old mice and rats were used for subcutaneous and subgingival transplantation, and 18-month-old beagles were used for alveolar bone augmentation. All experimental protocols were approved by the Institute for Laboratory Animal Research of the Ninth People's Hospital Affiliated with Shanghai JiaoTong University, School of Medicine (approval number: SH9H-2020-A227-1).

## Declaration of competing interest

The authors declare that they have no known competing financial interests or personal relationships that could have appeared to influence the work reported in this paper.

## Appendix A Supplementary data

Multimedia component 1 Additional supporting information, including supplementary figures and table, can be found in the Supplementary File.
