# Supplementary material for: An axolotl limb regeneration-inspired strategy to enhance alveolar bone regeneration
Source: Bioact Mater. 2025 Feb 19;48:242–56. doi: 10.1016/j.bioactmat.2025.02.020 (PMC11880770; doi:10.1016/j.bioactmat.2025.02.020)
Supplement: Multimedia component 1 — Additional supporting information, including supplementary figures and table, can be found in the Supplementary File. [file mmc1.docx]

**Supplementary Data** **for**

**An axolotl limb regeneration-inspired strategy to enhance alveolar bone regeneration**

Rongpu Liu *et al.*

Corresponding author: Wenjie Zhang, zhangwenjie586@126.com; Xinquan Jiang, xinquanjiang@aliyun.com

**This PDF file includes:**

Table S1

Figs. S1 to S4

| Name | Semi-open silk sponge | | No-channel/channel array PDMS film | |
| --- | --- | --- | --- | --- |
| Group | Epidermis | Muscle | Flat | Channel |
| Model | Subcutaneous transplantation | | Subcutaneous transplantation | |
| Schematic | 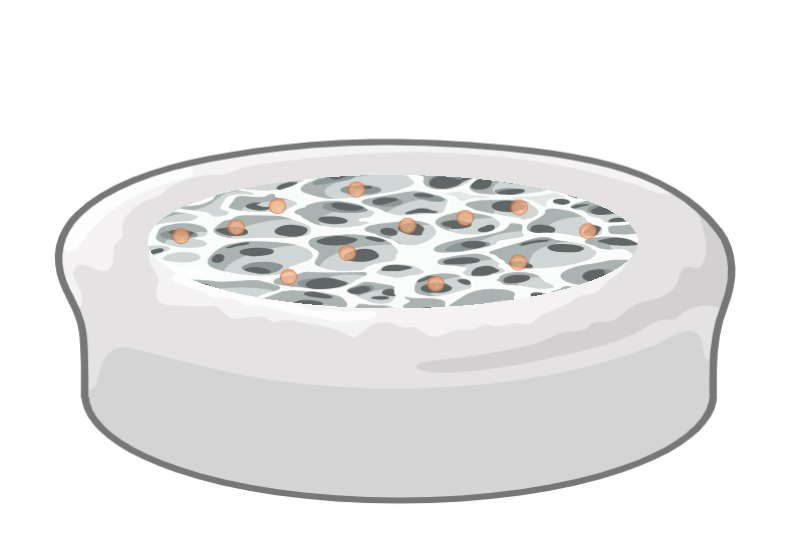 | | 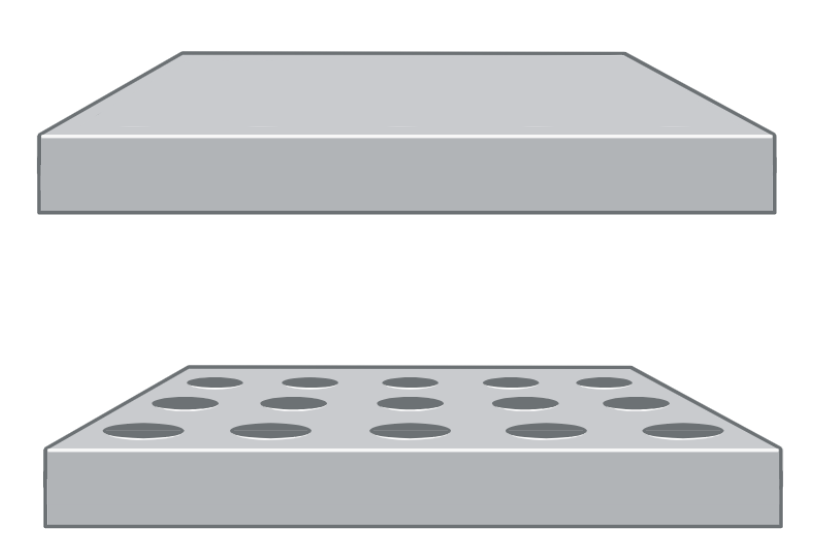 | |
| Dimensions (mm) | 5 (diameter) x 3 (height) | | 10 (lenth) x 6 (width) x 1(height) | |
| BMP-2 loading method | Cryogenic freeze-drying adsorption in gelatin | | / | |
| Name | Single-channel PDMS film | | PDMS cube | |
| Group | Control | BMP-2 | Control | BMP-2 |
| Model | Subcutaneous transplantation  Subgingival transplantation | | Subcutaneous transplantation | |
| Schematic | 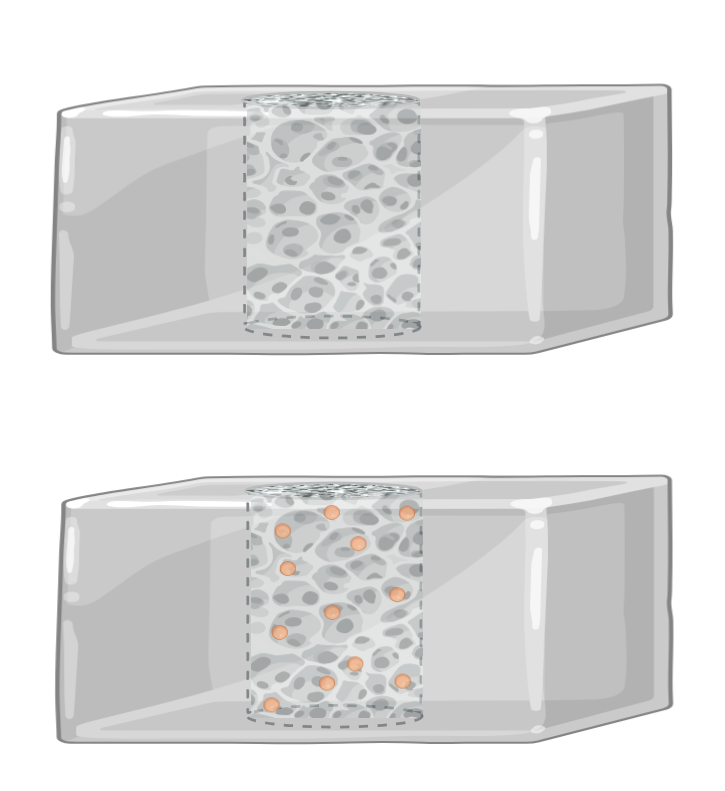 | | 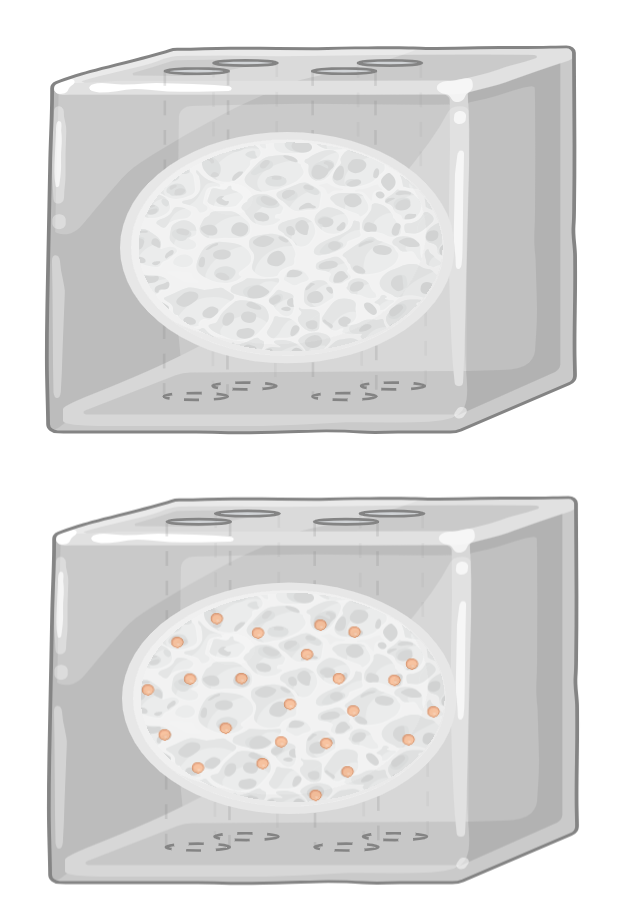 | |
| Dimensions (mm) | 5 (lenth) x 3 (width) x 2 (height) | | 8 (lenth) x 8 (width) x 6 (height) | |
| BMP-2 loading method | Cryogenic freeze-drying adsorption in silk | | Cryogenic freeze-drying adsorption in silk | |

Table S1.

**Table S1.** **PDMS-based composites fabricated in this study.**

Fig. S1.


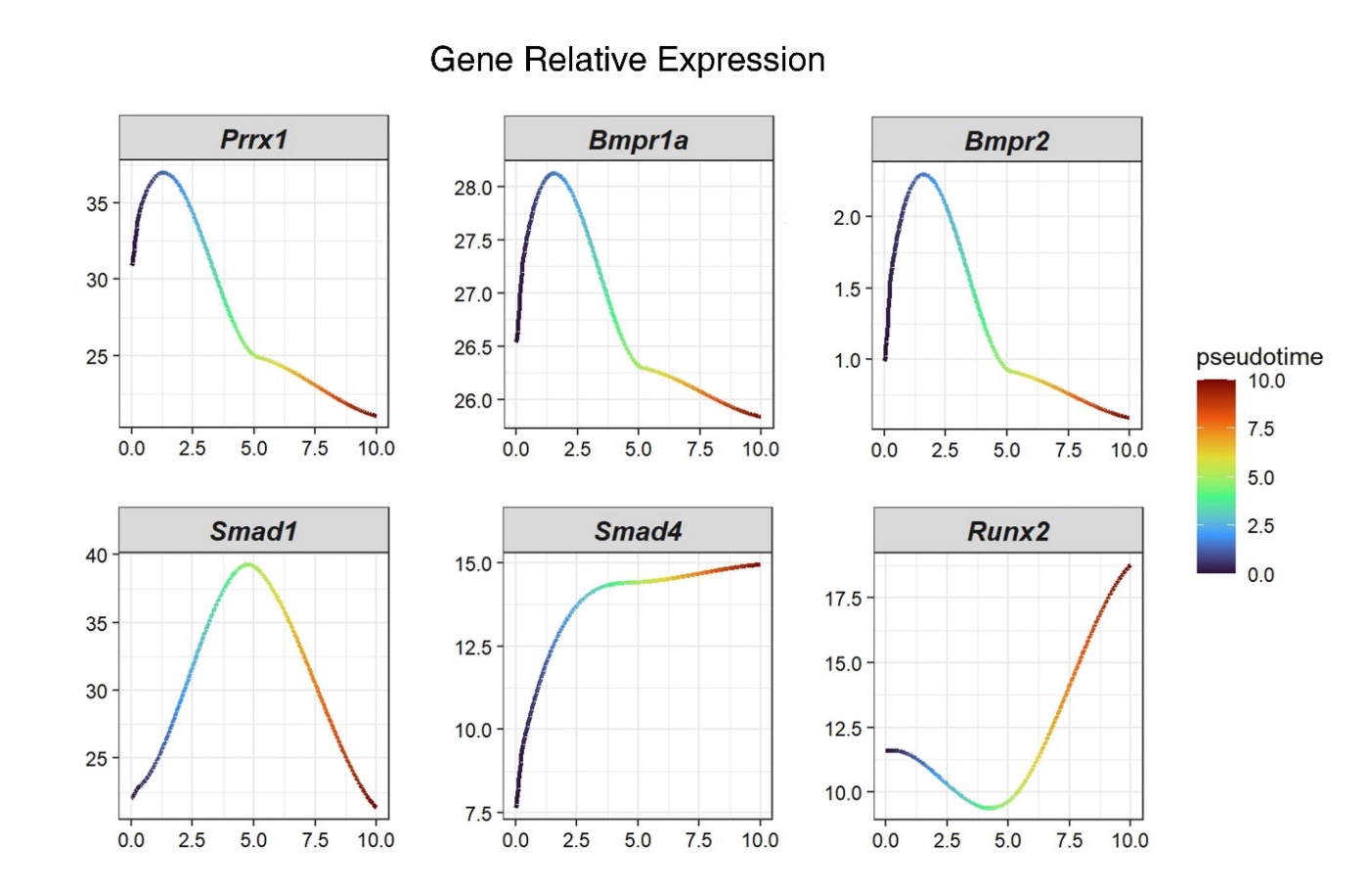


Fig. S1. Expression patterns of Prrx1 and BMP signaling pathway-associated genes.


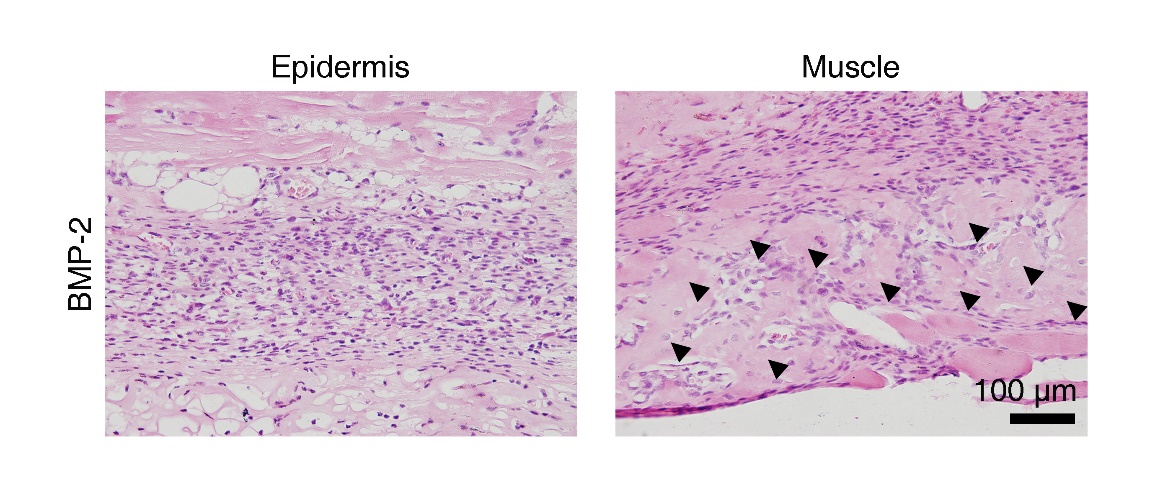
Fig. S2.

Fig. S2. HE staining of semi-open silk sponges subcutaneously implanted with open sides facing either the epithelial or muscular region. Black arrows indicate the matrix secreted by osteoblasts.


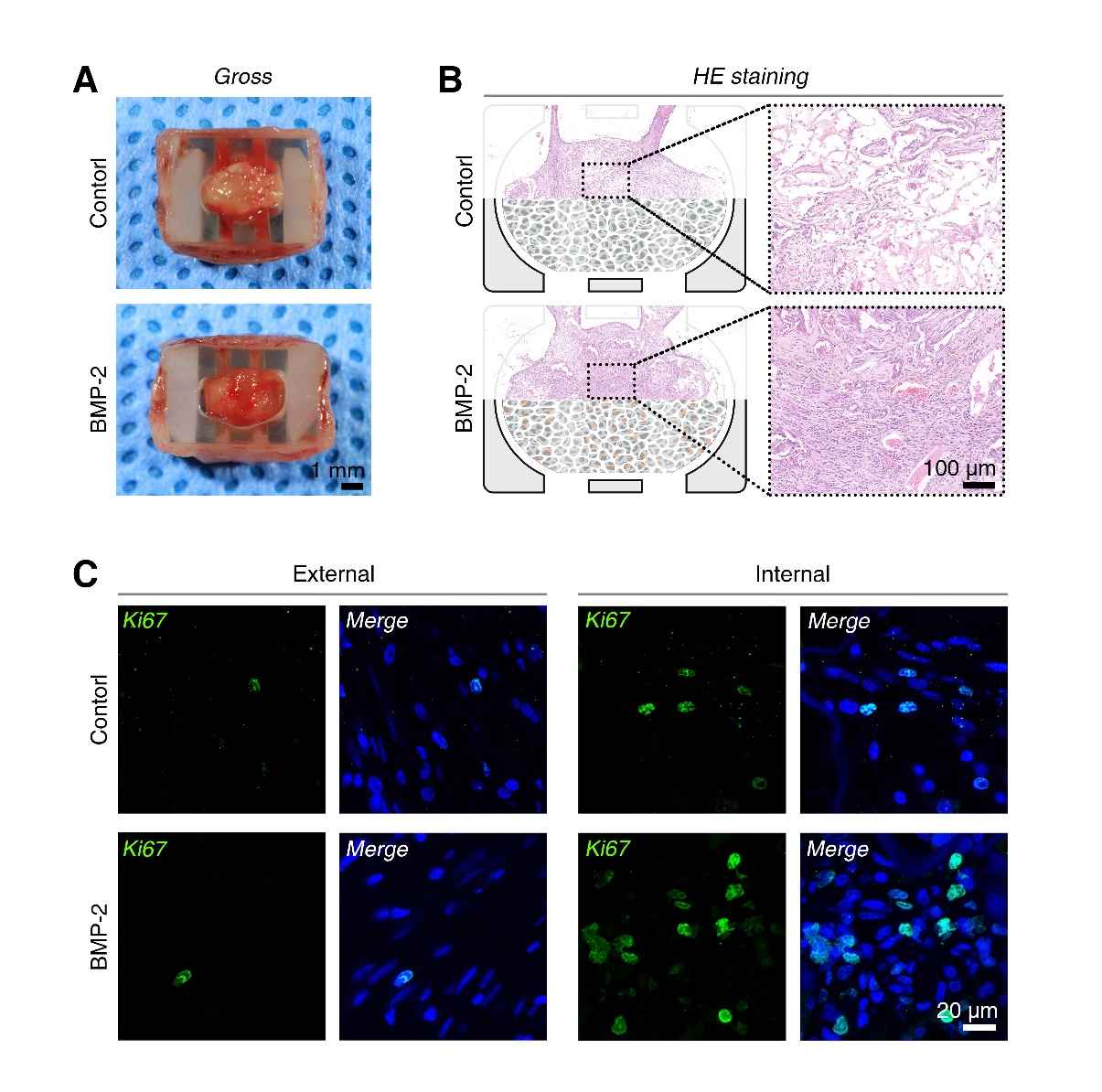
Fig. S3.

Fig. S3. BMP-2 promotes rapid infiltration of vascularized soft tissue within PDMS cubes. (A and B) Macroscopic observations (A) and HE staining (B) showing ingrowth of vascularized soft tissue. (C) Ki67 immunofluorescence staining to detect proliferative cells in the external and internal regions of PDMS cube.


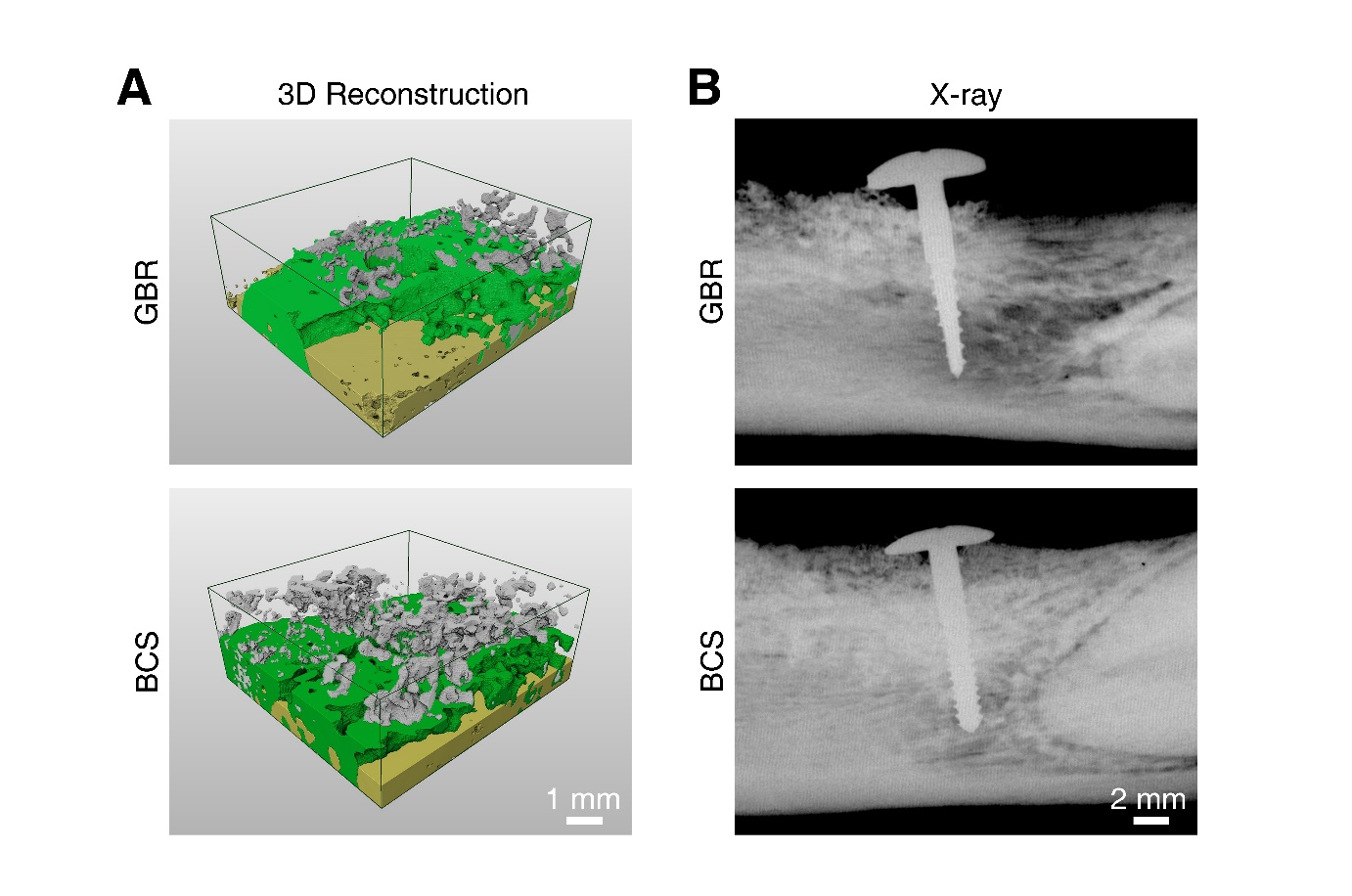
Fig. S4.

Fig. S4. Tenting screw BCS maintains osteogenic space stability in mandibular defect. (A) Layered 3D reconstruction visualizing the soft tissue, bone tissue, and bone substitutes surrounding the tenting screws. (B) X-ray imaging showing integration between bone substitutes and alveolar bone.
